# Effect of sugar beet tubers as a partial replacer to green fodder on production performance and economics of lactating Surti buffaloes in lean period

**DOI:** 10.14202/vetworld.2015.15-18

**Published:** 2015-01-06

**Authors:** L. M. Sorathiya, M. D. Patel, K. K. Tyagi, A. B. Fulsoundar, A. P. Raval

**Affiliations:** Livestock Research Station, Navsari Agricultural University, Navsari, Gujarat, India

**Keywords:** economics, production performance, sugar beet and Surti buffalo

## Abstract

**Aim:**

The objective of this study was to evaluate the effects of sugar beet tubers as a replacer to green fodder on production performance and economics of lactating Surti buffaloes.

**Materials and Methods:**

This trial was conducted at the Livestock Research Station, Navsari Agricultural University, Navsari. Twenty lactating Surti buffaloes in a changeover experimental design were selected to assess the effects of replacing green fodder with sugar beet (*Beta vulgaris* L.) tubers on production performance, economics of feeding sugar beet and blood biochemical profile. Half (50%) of the hybrid Napier was replaced with sliced sugar beet tubers in the ration of experimental animals.

**Results:**

Partial replacement of hybrid Napier with that of sugar beet tubers numerically improved dry matter intake, milk yield, 4% fat corrected milk and milk composition parameters such as fat, solid non-fat, protein and lactose, but not significantly. The blood parameters were in normal range and non-significant except that of glucose and triglycerides, which were increased in the sugar beet group. Replacing sugar beet tubers also proved to be cost-effective with improved net profit around Rs. 6.63/day.

**Conclusion:**

It can be concluded that 50% hybrid Napier fodder can be replaced with sugar beet tubers without any adverse effect on animal production performance, milk composition blood biochemical profile and economics of feeding.

## Introduction

The shortage of green fodder particularly in summer is one of the major constraints for dairy development in India. Majority of energy feed ingredients are now trading in the commodity market; hence, their price has dramatically increased in the recent years. This hike in the feed prices encouraged the nutritionist to search cheaper high energy feed ingredients [1].

Sugar beet (*Beta vulgaris* L.) is a promising high yielding summer crop that can be grown well in the coastal area. It has several advantages over traditional fodder crops that it can tolerate the soil salinity up to 5 Salinity of the saturation extract (ECe). It’s total yield, above and under the ground, can directly be used in animal feeding. The sugar beet top can also be used for silage making. The roots can also be stored in the soil without being greatly damaged, thus used when needed. Therefore, cultivation may help in overcoming the problem of shortage of green fodder in summer season [2,3]. However, very little research has been carried out on the comparative feeding value of sugar beet feeding in India. As it is new fodder crop, its potentiality and suitability in animal feeding should be determined.

Hence, present trial was conducted in which 50% hybrid Napier grass was replaced with improved variety of sugar beet tubers (Variety - PAC- 60008, Seasvanderhave, Belgium) in lactating Surti buffaloes in order to study their effect on production performance and economics.

## Materials and Methods

### Ethical approval

The experiment was sanctioned by Institutional Animal Ethical Committee of Veterinary College, Navsari Agricultural University, Navsari.

### Study area

The present experiment was conducted on twenty (n=20) lactating Surti buffaloes at Livestock Research Station, Navsari, Gujarat during the summer season in 2013.

### Animal shed

The experiment was conducted in the southern side of semi-loose, head to head and pakka shed located at the Livestock Research Station, Navsari Agricultural University, Navsari. The area of the southern part of shed was 25 × 16 m enclosed by 1.5 m high brick wall with pakka concrete floor. There was continuous manger having 90 cm width with 30 cm height in shed. The experimental animals were tied with galvanized iron chain in a row by keeping safe distance between animals to avoid sharing of feeds and fodders.

### Experimental animals

Twenty mid lactating Surti buffaloes of 1-4 parity were randomly divided into two homogeneous equal groups, ten in each group. The experiment was conducted in two phases of 3 weeks each by using the switch over design [4].

### Animal feeding

Control group (n=10) was fed isonitrogenous (12.5% CP) basal diet and the treatment group (n=10) fed isonitrogenous basal diet along with sugar beet tubers, meeting the nutrient requirement of lactating Surti buffaloes [5]. A half (50%) of hybrid Napier fodder in the treatment ration was replaced with sliced sugar beet tubers on dry matter (DM) basis. Relative proportions of various feed ingredients offered and its proximate composition are presented in Table-1. Before starting actual experiment and during the change of phase 1-week pre-experimental period/change over phase was followed. All the experimental buffaloes were fed with a mixture of chaffed green and dry fodder at 10 am in morning and 3 pm afternoon. Concentrate was offered as per proportion twice daily during milking. Water was provided *ad libitum*. The sugar beet tubers were sliced manually by knifes and weighed quantity was offered to the experimental group in the morning at 11.30 am.

**Table-1:** Proximate composition (% on DM basis) and proportion of feed ingredients used in experiment.

Ingredient	Proportion (%)		OM	CP	CF	EE	NFE	Ash

	Control	Treatment						
Concentrate	25	19	88.50	20.00	10.48	2.81	45.21	11.50
Cotton seed cake	7	13	94.80	30.60	21.40	9.10	33.70	5.20
Hybrid napier	28	14	87.31	9.48	31.77	1.83	44.23	12.69
Paddy straw	40	37	81.35	6.78	29.24	2.01	43.32	18.65
Sugar beet tubers	0	17	94.90	5.20	12.30	1.60	74.80	6.10

NFE=Nitrogen free extract, EE=Ether extract, CF=Crude fiber, CP=Crude protein, DM=Dry matter

### Data collection

Average daily feed offered and left over were accurately measured in order to determine average DM intake (DMI). Daily milk yield was recorded in morning and evening then it was averaged. Fat corrected milk (FCM) at 4% fat content was derived by using the standard method [6]. The milk composition, i.e. fat, solid non-fat (SNF), protein, lactose was measured using Automatic milk analyzer (Lactoscan^®^). The feed cost was calculated by considering prevailing market price of feed ingredients used in the experiment. The market price of milk was calculated based on their average fat content (rs. 540/kg fat). Daily income generated from milk production was obtained by multiplying average daily milk yield (kg) with the market price of milk. The net profit was calculated by deducting feed cost from daily income of milk production. The effect of sugar beet feeding on blood biochemical parameters was also ascertained. Blood samples were collected by jugular vein puncture from all buffaloes twice at the end of each phase, into plain vacutainer tubes for serum separation for biochemical and enzyme assay. Simultaneously blood samples were collected in blood collection tubes containing anticoagulant and sodium fluoride for glucose determination. Serum and plasma were separated and preserved at −80°C until analysis. The various biochemical and enzymes were analyzed on semi-automatic biochemical analyzer (Merck) by using commercial kits of Randox limited as per procedure described in kit inserts.

### Statistical analysis

The data were tabulated and analyzed using IBM^®^ SPSS^®^ statistics version 20.

## Results and Discussion

### DMI

DMI (Kg/d) was slightly increased from 10.97±0.11 in control to 11.12±0.11 in the treatment but remained non-significant (Table-2). However, DMI increase in the treatment group might be associated with better palatable and more NDF content of sugar beet. Earlier work on lactating cows also revealed that DMI remained statistically similar (control: 10.1 kg vs. treatment: 10.6 kg) with replacement of steam-flaked corn with dried shredded sugar beet in cattle in California [7,8]. Statistically similar DMI (0.97 kg in control vs. 0.85 kg in treatment) with sugar beet supplementation was also found in experiment conducted on Sudanese desert lamb in Sudan [9]. In contrast, several studies found increased DMI with Sugar beet replacement [10,11].

**Table-2:** Effect of sugar beet tubers feeding on milk production and composition in Surti buffaloes.

Parameter	Control	Treatment	p value
DMI (kg/d)	10.97±0.11	11.12±0.11	0.359
Milk Production (kg/d)	5.90±0.54	6.00±0.59	0.618
4% FCM (kg/d)	8.54±0.21	8.67±0.21	0.452
Milk composition (%)			
Fat	6.84±0.10	6.99±0.10	0.273
SNF	9.79±0.04	9.80±0.04	0.828
Protein	3.84±0.02	3.86±0.02	0.579
Lactose	4.91±0.03	4.89±0.03	0.547
Economics			
Price/kg of milk (Rs. 540/Kg fat)	36.88	37.75	
Milk Produced (Rs/d) (serial No. 2×serial No. 8)	217.59	226.50	
Cost of feeding (Rs./d)	111.25	113.53	
Net profit/Animal/d (Rs.)	106.34	112.97	
Increase in net profit Rs./Animal/d		6.63	

DMI=Dry matter intake, SNF=Solid non-fat, FCM=Fat corrected milk

### Production performance

Milk yield (Kg/d) was increased slightly in buffaloes fed sugar beets; however, it was statistically (p≥0.05) similar with control. Literature also revealed that sugar beet increased milk production numerically (up to 0.5 kg/day), but not significantly and slight increase in milk production might be due higher water and sugar content of sugar beet [12]. Milk fat percentage and 4% FCM of control group were 6.99±0.10, 8.67±0.21 and treatment group were 6.83±0.10, 8.54±0.21, respectively remained statistically (p≥0.05) similar which was in accordance with results reported earlier in lactating Holstein-Friesian cows in Ireland, UK [13]. Other milk composition parameters like SNF, protein, and lactose (Table-2) also remained non-significant are in agreement with the findings of one earlier experiment [14]. Possible reasons for a slight increase in milk fat and alteration in other milk parameter, i.e. milk lactose might be due to the high concentration of neutral detergent soluble fiber, especially pectin in sugar beet. Further, pectin fermentation produces less lactate and a higher ratio of acetate to propionate without affecting cellulose and hemicellulose digestion [15]. These results have clearly demonstrated that sugar beet feeding to lactating buffaloes did not have any adverse effect on milk production or milk composition.

### Economics of feeding sugar beet tubers

Relative market price (Rs/kg) of various feed ingredients i.e., concentrate, cottonseed cake, hybrid Napier, paddy straw and sugar beet were 14.5, 17.5, 2, 2 and 2, respectively. Cost of feeding in both the group was almost similar. The economics of sugar beet feeding (Table-2) revealed that the net profit of feeding sugar beet to lactating Surti buffaloes was Rs. 6.63 higher than the control group. The present findings are in agreement with the findings of experiments conducted on cattle [16], sheep and goats [9,10 and 17]. This finding clearly shows that sugar beet is not increasing the feeding cost.

### Blood biochemical profile

Majority of blood parameters remained unchanged and in their normal range with sugar beet supplementation except with that of glucose and triglycerides (Table-3). The lack of treatment effect on biochemical parameters in our study are consistent with the report [18] in which there was no change in blood parameters when barley was replaced with SBP for fattening lamb. Glucose and triglycerides level increased significantly (p≤0.05) with the sugar beet supplementation, this might be due to feeding of readily hydrolysable sugar content in sugar beet [19]. In contrast, plasma glucose concentration decreased in lactating dairy cows when sugar beet pulp content was increased at the expense of corn [20] and oat [21]. This result may be attributed to lower production of propionate in the rumen [19].

**Table-3:** Blood biochemical parameter of experimental Surti buffaloes.

Parameters	Control	Treatment	p value
Glucose (mg/dl)	50.78^b^±1.20	52.06^a^±1.28	0.001^**^
Triglycerides (mg/dl)	45.45^a^±2.92	41.56^b^±2.98	0.054^*^
Cholesterol (mg/dL)	38.84±1.41	40.35±1.36	0.074
Albumin (g/dL)	3.08±0.10	3.10±0.11	0.110
Total protein (g/dL)	7.97±0.27	7.67±0.28	0.115
Calcium (mg/dL)	8.88±0.20	8.86±0.19	0.793
Phosphorus (mg/dL)	6.25±0.25	6.76±0.26	0.120
Magnesium (mg/dL)	3.30±0.19	3.22±0.18	0.796
ALT (u/L)	38.54±2.28	39.95±2.20	0.770
AST (u/L)	71.27±3.40	69.03±3.13	0.410
Creatinine (mg/dL)	1.44±0.06	1.41±0.06	0.794
Urea (mg/dL)	35.57±0.81	36.52±0.88	0.396

Means with different superscript in a row differ significantly

* p<0.05,

** p<0.01,

ALT=Alanine aminotransferase, AST=Aspartate aminotransferase

Blood urea level decreased in the treatment as compared to control, this might be due to partial reductions in provision of ruminal ammonia for subsequent hepatic conversion to urea [22]. Sugar beet pulp (SBP) inclusion (33%) in ration of steers found significantly (p<0.01) decreased plasma urea nitrogen than control steers fed with no SBP (21 vs. 16.25 mg/dl) [15].

## Conclusion

It can be concluded that 50% hybrid Napier fodder can be replaced with sugar beet tubers without any adverse effect on animal production performance, milk composition blood biochemical profile and economics of feeding. This would lead to encouragement of sowing sugar beet, especially in south Gujarat to participate in solving of green fodder shortage during the summer season in coastal areas having problem of soil salinity.

## Author’s Contributions

LMS was the principal investigator for this experiment. MDP contributed in blood biochemical analysis. ABF contributed valuable suggestions during experiment. KKT contributed in statistical analysis part. APR contributed as a consultant animal nutritionist. All authors read and approved the final manuscript.
